# Editorial: Sex differences in adiposity and cardiovascular disease

**DOI:** 10.3389/fgwh.2023.1177718

**Published:** 2023-06-19

**Authors:** Amol Janorkar, Rodrigo O. Marañón

**Affiliations:** ^1^ Department of Biomedical Materials Science, University of Mississippi Medical Center, Jackson, MS, United States; ^2^ Department of Physiology, Faculty of Medicine, Universidad Nacional de Tucuman, San Miguel de Tucumán, Argentina; ^3^ National Council on Scientific and Technical Research (CONICET), Tucuman, Argentina

**Keywords:** adiposity, sex differences, cardiovascular disease, women health, sex hormones

**Editorial on the Research Topic** Sex differences in adiposity and cardiovascular disease

The Research Topic *Sex Differences in Adiposity and Cardiovascular Disease* was proposed a few months before the COVID pandemic by AJ from the University of Mississippi Medical Center (USA) and ROM from the Universidad Nacional de Tucuman (Argentina) with the goal to aid the understanding of the role of the body fat constitution as a cardiovascular risk factor, and whether this risk is similar for men and women. In addition to this, to determine if sex steroids play a specific role either in the health of females and males.

Today, although there is progress in the study of the effect of sex hormones on the adipocyte’s metabolism and distribution, its role is still unclear in cardiovascular and metabolic diseases. Controversial evidence suggests that different fat types contribute to the high risk of cardiovascular events (1). Thus, visceral fat is meant to contribute to a deleterious cardiovascular effect, while subcutaneous fat has the opposite function (2). In this hypothesis, sex hormones may play a vital role, increasing or decreasing the fat mass in the mentioned depots and contributing to the fat metabolism’s equilibrium. However, the role of androgens in females, and estrogens in males, need further investigation.

In our proposal, we received six original research manuscripts that indirectly fill the aim of our topic and contribute to the leading journal Frontiers in Global Women’s Health. Of 100% of the manuscript submitted to our topic, 67% were accepted, and 33% were rejected. The case-control study by Zhao et al. examines the association between hypertension and its control with atrial fibrillation and how the biological sex affects this association. The original research study by Khatami et al. explored whether iron biomarkers mediate sex differences in NT-proBNP levels. Authors investigated, using linear regression analyses, the association of sex and iron biomarkers with NT-proBNP levels, independent of adjustment for potential confounders. Another original research study by Li et al. examined the effects of age and sex on outcomes of coronary artery bypass grafting (CABG) and percutaneous coronary intervention (PCI) in non-ST-segment elevation acute coronary syndrome (NSTE-ACS) patients with the three-vessel disease (TVD) and the interaction between treatment and age or sex in NSTE-ACS and TVD. Finally, the cohort study by Huang et al. showed the sex difference temporal trends of short-term and long-term mortality after CAD admission in China. It was based on 11 years of data from a sample of 24,432 people in the largest cardiovascular disease center in South China.

In this experience, we learned to manage different situations involved during the publication process, where, in the present day, the offer to publish and review manuscripts is increasing. The limitation was finding reviewers for the specific field.
